# Parkinson's Disease-Related Protein, α-Synuclein, in Malignant Melanoma

**DOI:** 10.1371/journal.pone.0010481

**Published:** 2010-05-05

**Authors:** Yasuhiro Matsuo, Tetsu Kamitani

**Affiliations:** Department of Medicine, Center for Molecular Chaperone/Radiobiology and Cancer Virology, Cancer Center, Medical College of Georgia, Augusta, Georgia, United States of America; Brigham and Women's Hospital, Harvard Medical School, United States of America

## Abstract

**Background:**

Melanoma is the major cause of skin cancer death worldwide. Parkinson's disease is a neurodegenerative disorder that is caused by mutation of α-synuclein or other genes. Importantly, epidemiological studies have reported co-occurrence of melanoma and Parkinson's disease, suggesting that these two diseases could share common genetic components.

**Methodology/Principal Findings:**

Recently, we found that human melanoma cell lines highly express α-synuclein, whereas the protein is undetectable in the non-melanoma cancer cell lines tested. To investigate the expression of α-synuclein in human melanoma tissues, we immunostained sections of melanoma, nevus, non-melanocytic cutaneous carcinoma, and normal skin. α-Synuclein was positively detected in 86% of the primary and 85% of the metastatic melanoma sections, as well as in 89% of nevus sections. However, α-synuclein was undetectable in non-melanocytic cutaneous carcinoma and normal skin.

**Conclusions/Significance:**

The Parkinson's disease-related protein, α-synuclein, is expressed in both malignant and benign melanocytic lesions, such as melanomas and nevi. Although α-synuclein cannot be used to distinguish between malignant and benign melanocytic skin lesions, it might be a useful biomarker for the diagnosis of metastatic melanoma.

## Introduction

Malignant melanoma is the major cause of skin cancer mortality, and its incidence is rising worldwide [1], [2]. Clinical and histological variables such as primary tumor invasion, ulceration, and lymph node status might fail to identify early stage disease that will eventually progress [3]. Therefore, an accurate histological diagnosis is only the first step towards a rational and effective treatment for melanoma [2]. Histological biomarkers might help us to diagnose and identify patients with early-stage melanoma who are likely to develop advanced disease and would benefit from additional therapies [3]. S-100, HMB-45, and MART-1 are well known biomarkers for melanoma, and antibodies against these molecules have been used for immunostaining to diagnose malignant melanoma [4].

Specifically, the anti-S-100 antibody demonstrates excellent sensitivity because the S-100 protein is expressed in almost all primary and metastatic malignant melanomas. Unfortunately, however, S-100 has limited specificity; it is expressed in many other malignant tumors [4]. HMB-45 and MART-1 are melanocyte-specific proteins and are most commonly used as melanocytic markers. Although HMB-45 is highly specific to melanoma, the clinical utility of HMB-45 is limited because it is not expressed by 20% to 40% of metastatic melanomas [4], [5], [6]. MART-1 has been reported to be more specific and more sensitive than HMB-45 in both primary and metastatic melanoma [4], [7], [8]. MART-1 has also been demonstrated to have a higher diagnostic accuracy than S-100 and HMB-45 staining [4], [9]. Thus, MART-1 currently seems to be the most useful histological biomarker for the diagnosis of melanoma.

Recently, epidemiological studies have suggested a link between melanoma and Parkinson's disease (PD), a neurodegenerative disease associated with mutations of α-synuclein [10], [11], [12]. α-Synuclein is a highly charged 140-amino-acid protein that is predominantly expressed in central nervous system neurons, where it is localized around synaptic vesicles in presynaptic terminals [13]. Although the function of α-synuclein is still unclear, genetic studies have demonstrated that point mutation or amplification of the α-synuclein gene can cause neurodegenerative disorders, termed α-synucleinopathies, such as PD and dementia with Lewy bodies (DLB) [13]. Thus, α-synuclein is involved in the pathogenesis of neurodegenerative disorders, but its relationship with melanoma remains unknown.

In this study, we demonstrate that α-synuclein is highly expressed in melanoma cell lines, both primary and metastatic melanoma tissues, and nevus tissues.

## Materials and Methods

### Cell Lines and Culture Conditions

We purchased the following human cell lines from American Type Culture Collection (Manassas, VA): SH-SY5Y, SK-MEL28, K562, HEK293, HeLa, HT1080, HL60, SW620, U937, U2OS, SK-NSH, Daoy, Y79, A-375, MeWo, and WM266-4. BJAB and U251MG were generous gifts from Dr. Fred Wang (Harvard Medical School) and Dr. Yoshiki Saito (M. D. Anderson Cancer Center), respectively. K562, HL60, U937, Y79, and BJAB cells were maintained in RPMI 1640 medium supplemented with 10% fetal calf serum and antibiotics. Other cell lines were maintained in Dulbecco's modified Eagle's medium supplemented with 10% fetal calf serum and antibiotics.

### Antibodies

Mouse monoclonal anti-α-synuclein antibodies 4D6 and 7B12.2 were purchased from Santa Cruz Biotechnology (Santa Cruz, CA) and Chemicon (Temecula, CA), respectively. Mouse monoclonal anti-HMB-45 antibody was purchased from Santa Cruz Biotechnology. Rabbit monoclonal anti-MART-1 antibody was purchased from Epitomics (Burlingame, CA). Rabbit polyclonal anti-actin antibody was purchased from Sigma (St. Louis, MO).

### Western Blot Analysis

Protein samples were treated for 1 h at 50°C in 2% SDS treating solution. After SDS-PAGE, Western blot analysis was performed according to the protocol provided with the ECL detection system (Amersham Pharmacia Biotech). Horseradish peroxidase-conjugated anti-mouse IgG or anti-rabbit IgG antibody (Santa Cruz Biotechnology) was used as a secondary antibody.

### Immunohistochemistry

To immunostain human tissues of malignant melanoma, nevus, non-melanocytic cutaneous carcinoma (squamous cell carcinoma and basal cell carcinoma), and normal skin, tissue microarray slides were purchased from Imgenex (San Diego, CA) and US Biomax (Rockville, MD). Slides were incubated in an oven at 60°C for 30 min, deparaffinized, and pretreated with Target Retrieval solution (Dako, Carpinteria, CA). Slides were next incubated with anti-α-synuclein antibody 4D6 at dilution 1∶100 for 1 h at room temperature, followed by washing with PBS. Slides were then incubated with alkaline phosphatase-conjugated anti-mouse IgG (Jackson ImmunoResearch Laboratories, West Grove, PA) for 1 h at room temperature and rinsed with PBS. Bound antibody was detected with Liquid Permanent Red substrate (Dako). Slides were then counterstained with hematoxylin (Richard-Allan Scientific, Kalamazoo, MI) and mounted. The slides were examined with an Axio Imager M1 microscope (Zeiss).

### Double Immunostaining for α-Synuclein and MART-1

A tissue microarray slide (Imgenex) was incubated in an oven at 60°C for 30 min. The slide was then deparaffinized and pretreated with Target Retrieval solution (Dako). Next, the slide was incubated with mouse monoclonal anti-α-synuclein (4D6) antibody at a dilution 1∶100 and rabbit monoclonal anti-MART-1 (EP1422Y) antibody at a dilution of 1∶20,000 for 1 h at room temperature. After washing, the slide was labeled with Cy2-conjugated AffiniPure F(ab')_2_ fragment donkey anti-mouse IgG for α-synuclein and Cy3-conjugated AffiniPure F(ab')_2_ fragment donkey anti-rabbit IgG for MART-1 at a dilution of 1∶500 for 1 h at room temperature. These secondary antibodies were purchased from Jackson ImmunoResearch Laboratories. Finally, the slide was analyzed by an Axio Imager M1 microscope (Zeiss). The localization of α-synuclein was shown by the green fluorescence of Cy2. The localization of MART-1 was shown by the red fluorescence of Cy3. Their colocalization was examined by the merging of both fluorescent images.

## Results

### α-Synuclein and Melanoma Markers are Specifically Expressed in SK-MEL28 Cells

**Figure 1:**
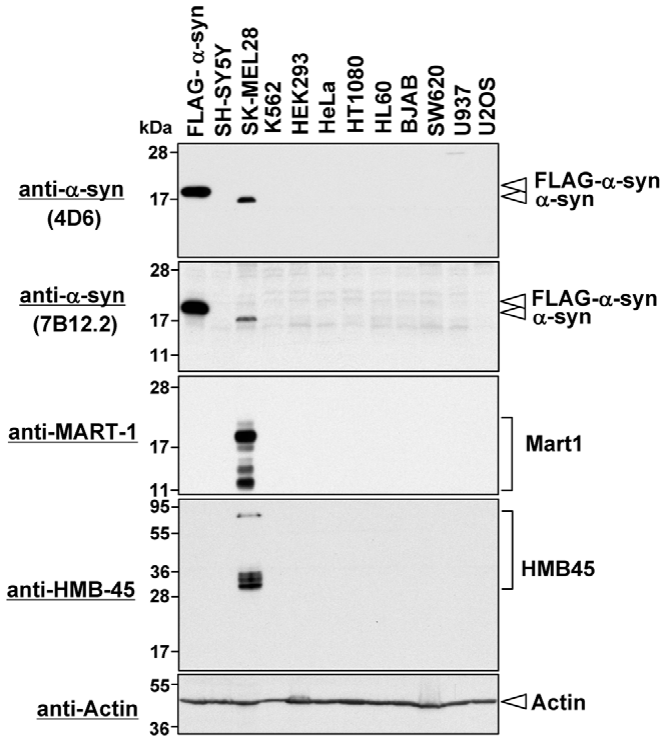
Expression levels of α-synuclein in human non-melanoma cell lines. Expression of endogenous α-synuclein was examined in different non-melanoma cancer cell lines by Western blotting using two different mouse monoclonal anti-α-synuclein antibodies (4D6 and 7B12.2). A total cell lysate of HeLa cells expressing FLAG-tagged α-synuclein was loaded as a positive control. To demonstrate expression of melanoma markers, we performed Western blotting using monoclonal antibodies against MART-1 and HMB-45. To demonstrate equal loading amounts of total cell lysates, Western blotting using anti-actin antibody was also performed (bottom panel). The detected proteins are indicated on the right. Molecular size markers are shown in kilodaltons.

Although α-synuclein is predominantly expressed in central nervous system neurons [13], which human cell lines highly express α-synuclein is not known. We therefore performed Western blotting to examine the expression levels of α-synuclein in 11 human cell lines: SH-SY5Y (neuroblastoma), SK-MEL28 (malignant melanoma), K562 (erythroleukemia), HEK293 (human embryonic kidney cell), HeLa (cervical adenocarcinoma), HT1080 (lung fibrosarcoma), HL60 (promyelocytic leukemia), BJAB (Burkitt's lymphoma), SW620 (colon adenocarcinoma), U937 (myeloid leukemia), and U2OS (osteosarcoma). As a positive control, we used HeLa cell transfectants expressing FLAG-tagged α-synuclein. As shown in Fig. 1, α-synuclein, as well as the melanoma markers MART-1 and HMB-45, was strongly expressed in the malignant melanoma SK-MEL28 cell line, but was undetectable in the other cell lines, suggesting that α-synuclein is specifically expressed in malignant melanoma SK-MEL28 cells.

### α-Synuclein Is Undetectable in Cell Lines of Brain Tumors and Retinoblastoma

**Figure 2:**
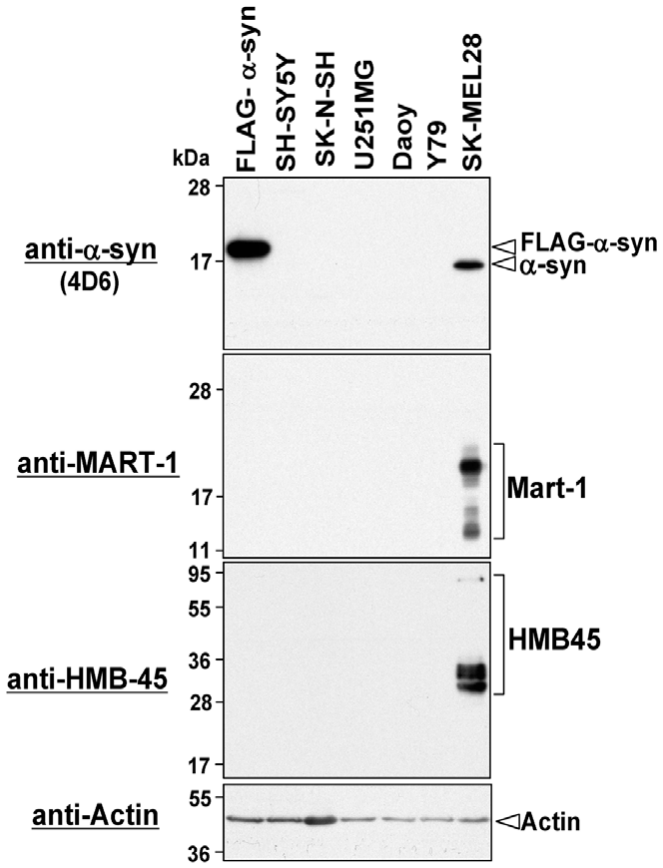
Expression levels of α-synuclein in cell lines of human brain tumors and retinoblastoma. Expression of endogenous α-synuclein was examined in cell lines of human brain tumors and retinoblastoma by Western blotting using mouse monoclonal anti-α-synuclein antibody 4D6. Total cell lysates of HeLa cells expressing FLAG-tagged α-synuclein and SK-MEL28 cells were loaded as positive controls. To demonstrate expression of melanoma markers, we performed Western blotting using monoclonal antibodies against MART-1 and HMB-45. To demonstrate equal loading amounts of total cell lysates, Western blotting using anti-actin antibody was also performed (bottom panel). The detected proteins are indicated on the right. Molecular size markers are shown in kilodaltons.

Since α-synuclein is predominantly expressed in the nervous system [13], we used Western blotting to examine its expression levels in human cancer cell lines derived from the nervous system, including the retinoblastoma Y79 cell line and 4 brain tumor cell lines, SH-SY5Y (neuroblastoma), SK-NSH (neuroblastoma), U251MG (glioblastoma), and Daoy (medulloblastoma). HeLa cell transfectants expressing FLAG-tagged α-synuclein and SK-MEL28 cells were used as positive controls. As shown in Fig. 2, the expression of α-synuclein was undetectable in the brain tumor and retinoblastoma cell lines tested.

### α-Synuclein Is Expressed in Some Melanoma Cell Lines

**Figure 3:**
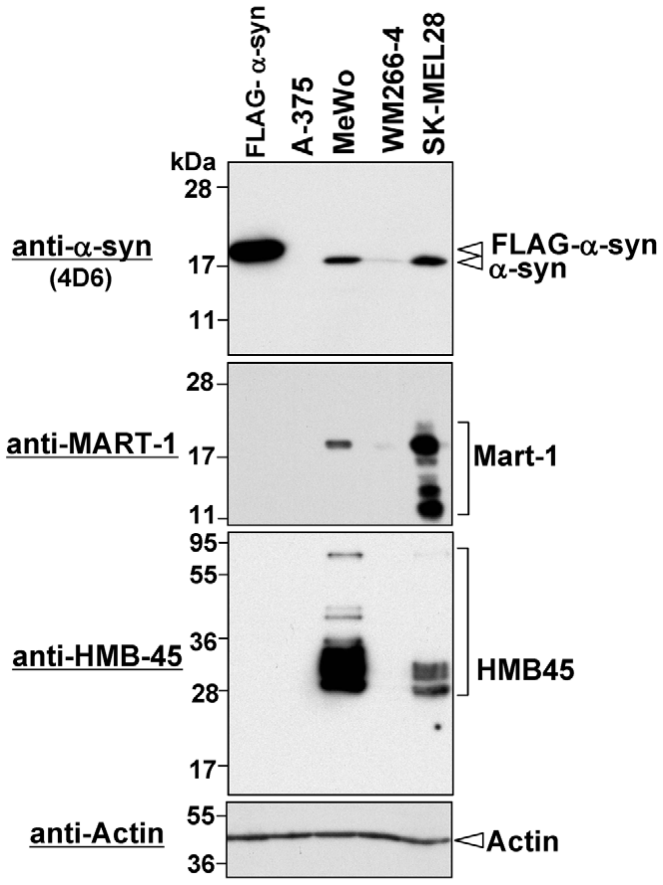
Expression levels of α-synuclein in human melanoma cell lines. Expression of endogenous α-synuclein was examined in melanoma cell lines by Western blotting using mouse monoclonal anti-α-synuclein antibody 4D6. Total cell lysates of HeLa cells expressing FLAG-tagged α-synuclein and SK-MEL28 cells were loaded as positive controls. To demonstrate expression of melanoma markers, we performed Western blotting using monoclonal antibodies against MART-1 and HMB-45. To demonstrate equal loading amounts of total cell lysates, Western blotting using anti-actin antibody was also performed (bottom panel). The detected proteins are indicated on the right. Molecular size markers are shown in kilodaltons.

α-Synuclein protein is highly expressed in the human malignant melanoma SK-MEL28 cell line, suggesting that it may be expressed in other melanoma cell lines. To test this possibility, we examined the expression levels of α-synuclein and two melanoma markers (MART-1 and HMB-45) in 3 other malignant melanoma cell lines, A-375, MeWo, and WM266-4, by Western blotting. HeLa cell transfectants expressing FLAG-tagged α-synuclein and SK-MEL28 cells were used as positive controls. As shown in Fig. 3, α-synuclein was strongly detected in MeWo and SK-MEL28 cells and weakly detected in WM266-4 cells, whereas it was undetectable in A-375 cells. Interestingly, both MeWo and SK-MEL28 were also positive for the melanosome-related marker proteins MART-1 and HMB-45, but A-375 was negative for them. WM266-4 was weakly positive for MART-1 and negative for HMB-45. These results suggest that α-synuclein is involved in melanosomes or melanogenesis.

### α-Synuclein Is Expressed in Malignant Melanoma Tissues

To examine the expression of α-synuclein in human malignant melanoma tissue samples, we immunostained 132 melanoma sections with antibody to α-synuclein. These tissue sections were derived from 98 primary lesions and 34 metastatic lesions. Surprisingly, 86% of the primary melanoma samples and 85% of the metastatic melanoma samples were positive for α-synuclein (Table 1).

**Table 1:** Summary of α-synuclein immunoreactivity in malignant melanoma.

	α-Synuclein	
	Negative	Positive
**Primary melanoma: 98**	14 (14%)	84 (86%)
**Metastatic melanoma: 34**	5 (15%)	29 (85%)
**Total: 132**	19 (14%)	113 (86%)

Lesions of primary melanoma (98 cases) include 84 skin, 4 eye, 3 anus, 2 nasal cavity, 2 rectum, and 3 vulva. Lesions of metastatic melanoma (34 cases) include 31 lymph nodes, 2 lungs, and 1 small intestine.

**Figure 4:**
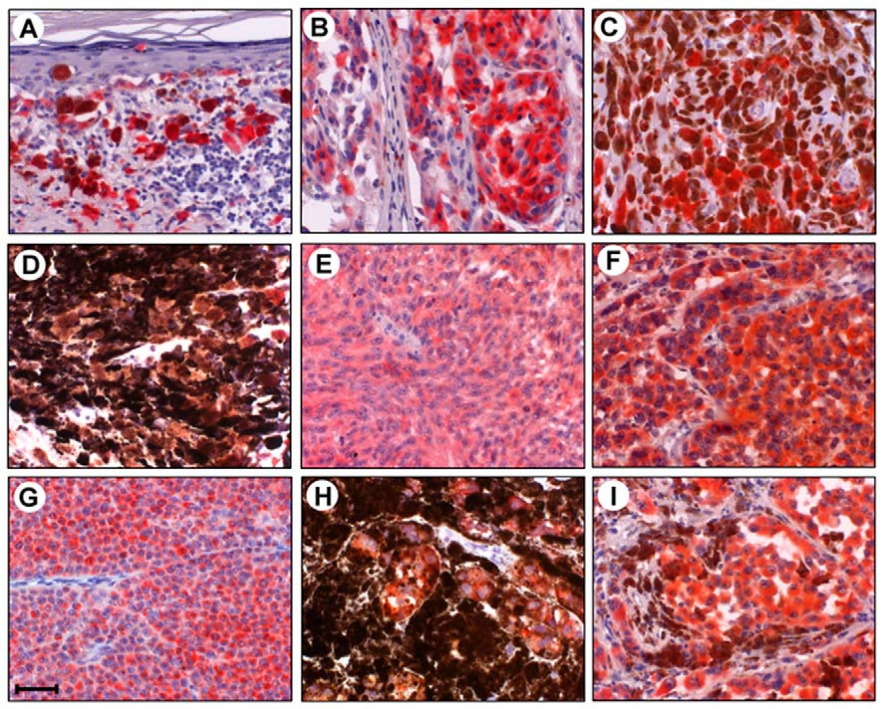
Expression of α-synuclein in human malignant melanoma tissues. Tissue sections of 132 melanoma samples were immunostained with mouse monoclonal anti-α-synuclein antibody 4D6 (see text for identification of samples). The sections were then incubated with an alkaline phosphatase-conjugated anti-mouse IgG. After washing, Liquid Permanent Red substrate was used to develop the reaction and detect α-synuclein-positive cells. The sections were then counterstained with hematoxylin and analyzed by microscopy. The localization of α-synuclein is shown by the red color of Liquid Permanent Red substrate. Nuclear counterstaining is shown by the blue color of hematoxylin. Melanin pigments are brown in melanoma cells. Scale bar indicates 50 µm.

Fig. 4 shows 9 of 132 samples of melanoma immunostainings. Panel A shows anti-α-synuclein immunostaining of primary melanoma (skin, face) from a 70-year-old female. α-Synuclein-positive, atypical melanoma cells are scattered in the dermis as well as infiltrating into the epidermis. The degree of pigmentation varies. Lymphocytic infiltration is present. Panel B shows immunostaining of primary melanoma (skin, trunk) from a 20-year-old female. There is nodular proliferation of melanoma cells in the nests without pigment production but with high expression of α-synuclein. Panel C shows immunostaining of primary melanoma (skin, left buttock) from a 55-year-old male. α-Synuclein-positive melanoma cells are proliferating in the dermis. Half of the melanoma cells generate melanin pigments. Vessel-like structures are observed. Panel D shows immunostaining of primary uveal melanoma (eyeball) from a 53-year-old male. Heavily pigmented melanoma cells can be seen. Some cells have less melanin pigments but express high levels of α-synuclein. Panel E shows immunostaining of primary melanoma (rectum) from a 38-year-old female. There is proliferation of spindle-shaped melanoma cells with α-synuclein expression but without pigment production. Panel F shows immunostaining of primary melanoma (anus) from a 77-year-old male. There is proliferation of melanoma cells with α-synuclein expression but without pigment production. Panel G shows immunostaining of metastatic melanoma (lymph node, right oxter) from a 44-year-old male. The lymph node is packed with round metastatic cells that express α-synuclein but do not produce melanin pigments. Panel H shows immunostaining of metastatic melanoma (lymph node, left groin) from a 72-year-old female. Metastatic melanoma cells are heavily pigmented in the lymph node. Some cells have less melanin pigments but express high levels of α-synuclein. Panel I shows immunostaining of metastatic melanoma (lung) from a 59-year-old male. Melanoma cells with α-synuclein expression but without brown melanin pigments are packed in the alveolar spaces, whereas melanoma cells with brown melanin pigments line on the alveolar walls. Nucleoli are prominent in melanoma cells.

Thus, α-synuclein can be immunostained in various types of malignant melanoma tissues.

### α-Synuclein Is Expressed in MART-1-negative Melanoma Cells

**Figure 5:**
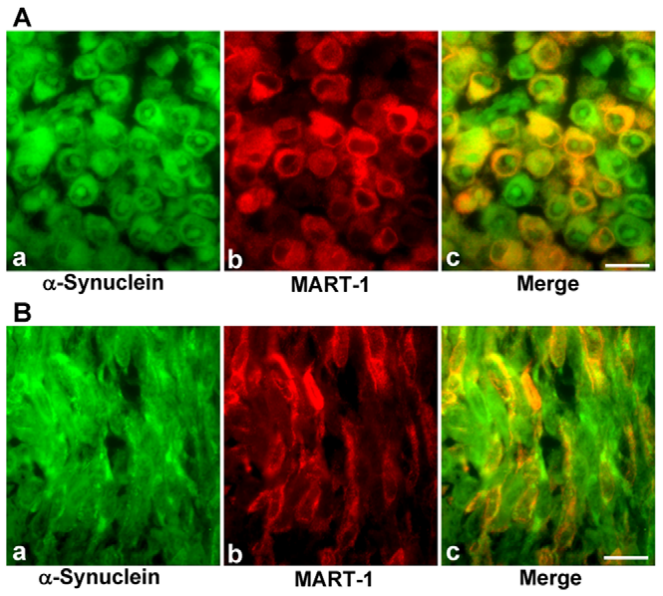
Relationship of α-synuclein with MART-1 in human malignant melanoma tissues. To investigate the relationship between α-synuclein and MART-1, tissue sections of melanoma samples (**A** and **B**) were double-immunostained with both mouse monoclonal anti-α-synuclein (4D6) antibody and rabbit monoclonal anti-MART-1 (EP1422Y) antibody. The sections were then labeled with Cy2-conjugated anti-mouse IgG for α-synuclein and Cy3-conjugated anti-rabbit IgG for MART-1. Finally, the sections were analyzed by fluorescence microscopy. The localization of α-synuclein is shown by the green fluorescence of Cy2 (panel **a**). The localization of MART-1 is shown by the red fluorescence of Cy3 (panel **b**). Their colocalization was examined by the merging of both fluorescent images (panel **c**). Scale bar indicates 20 µm.

MART-1 has currently been the most useful histological biomarker for malignant melanoma [4], [9]. To determine whether α-synuclein is more useful than MART-1 as a histological biomarker for melanoma, we double-immunostained 56 melanoma sections (derived from 42 primary lesions and 14 metastatic lesions) with both mouse monoclonal anti-α-synuclein antibody and rabbit monoclonal anti-MART-1 antibody (see Fig. 5 and Table 2). For primary melanoma, 86% of samples were positive for α-synuclein and 76% of samples were positive for MART-1. For metastatic melanoma, 86% of samples were positive for α-synuclein and 71% of samples were positive for MART-1 (Table 2). Thus, in comparison with MART-1, α-synuclein was detected in more cases of both primary and metastatic melanoma.

**Table 2:** Comparison between α-synuclein and MART-1 immunoreactivities in malignant melanoma.

	α-Synuclein		MART-1	
	Negative	Positive	Negative	Positive
**Primary melanoma: 42**	6 (14%)	36 (86%)	10 (24%)	32 (76%)
**Metastatic melanoma: 14**	2 (14%)	12 (86%)	4 (29%)	10 (71%)
**Total: 56**	8 (14%)	48 (86%)	14 (25%)	42 (75%)

Lesions of primary melanoma (42 cases) include 33 skin, 3 eye, 2 anus, 2 nasal cavity, and 2 rectum. Lesions of metastatic melanoma (14 cases) include 11 lymph nodes, 2 lungs, and 1 small intestine.

In Fig. 5A and B, we show 2 melanoma sections immunostained with both mouse monoclonal anti-α-synuclein antibody and rabbit monoclonal anti-MART-1 antibody. These sections were selected from 56 immunostained sections. Specifically, Fig. 5A shows double immunostaining of metastatic melanoma (lymph node) from a 47-year-old male. α-Synuclein is expressed in many round-shaped melanoma cells; both the cytoplasm and prominent nucleoli are immunostained (panel a). MART-1 is expressed only in the cytoplasm (panel b). Importantly, some α-synuclein-positive cells express MART-1 (shown by yellow and orange cells in panel c). However, other α-synuclein-positive cells do not (shown by green cells in panel c). Fig. 5B shows double immunostaining of primary melanoma (popliteal region) from a 56-year-old male. α-Synuclein is expressed in many spindle-shaped melanoma cells (panel a). MART-1 is expressed in the cytoplasm of some cells (panel b). Importantly, some α-synuclein-positive cells express MART-1 (shown by yellow and orange cells in panel c). However, other α-synuclein-positive cells do not (shown by green cells in panel c). Thus, α-synuclein is expressed in MART-1-negative melanoma cells in many cases.

### Melanoma Cells Highly Expressing α-Synuclein Generate No or Low Levels of Melanin Pigments

**Figure 6:**
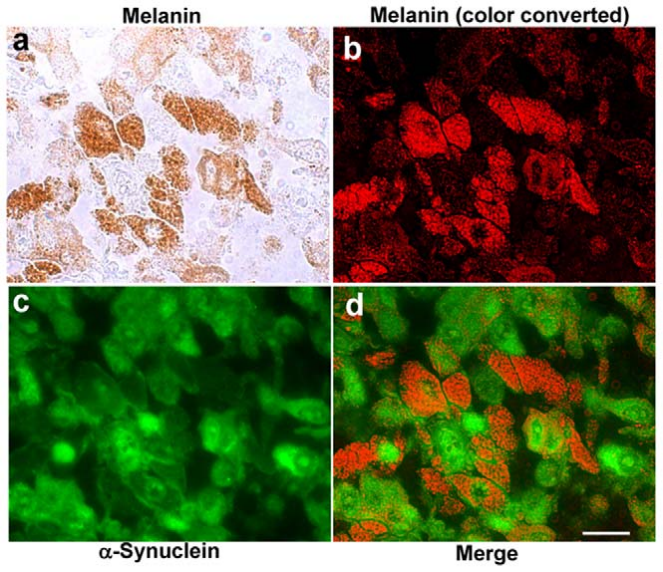
Relationship of α-synuclein with melanin pigments in human malignant melanoma tissues. To investigate the relationship of α-synuclein with melanin pigments, tissue sections of pigmented melanoma were immunostained with mouse monoclonal anti-α-synuclein antibody 4D6. The sections were analyzed by fluorescence microscopy to determine the localization of α-synuclein and by bright field microscopy to determine the localization of melanin pigments. The localization of α-synuclein is shown by the green fluorescence of Cy2 (panel **c**). The localization of melanin pigments is shown by the brown color in the bright field microscopy (panel **a**). The localization of melanin pigments is shown by the red color in the dark field (panel **b**) because the image of brown pigments (panel **a**) was inverted and the color was then converted to red (panel **b**). In panel **d**, the image of panel **b** is merged with panel **c**. Scale bar indicates 20 µm.

To investigate the relationship between the expression of α-synuclein and the generation of melanin pigments, we analyzed 17 tissue sections containing melanin-positive melanoma cells. Fig. 6 shows the α-synuclein immunostaining of metastatic melanoma (inguinal lymph node) from a 57-year-old female. We first acquired a bright field image to determine the localization of melanin pigments (panel a). The image of brown pigments was then inverted and the color was converted to red (panel b). As shown in panels a and b, almost half of the cells generate melanin pigments. Importantly, cells highly expressing α-synuclein show no or a very low level of melanin pigments (panels c and d). This finding was observed in all of the immunostained sections that are strongly positive for α-synuclein.

### α-Synuclein Is Expressed in Nevus Tissues

To examine the expression of α-synuclein in benign melanocytic lesions, we immunostained 19 nevus sections with antibody to α-synuclein. These tissue sections were derived from 6 compound nevus samples, 11 intradermal nevus samples, and 2 junctional nevus samples. As summarized in Table 3, 83% of the compound nevus samples, 91% of the intradermal nevus samples, and all samples of junctional nevus were positive for α-synuclein.

**Table 3:** Summary of α-synuclein immunoreactivity in nevi.

	α-Synuclein	
	Negative	Positive
**Compound nevus: 6**	1 (17%)	5 (83%)
**Intradermal nevus: 11**	1 (9%)	10 (91%)
**Junctional nevus: 2**	0 (0%)	2 (100%)
**Total: 19**	2 (11%)	17 (89%)

**Figure 7:**
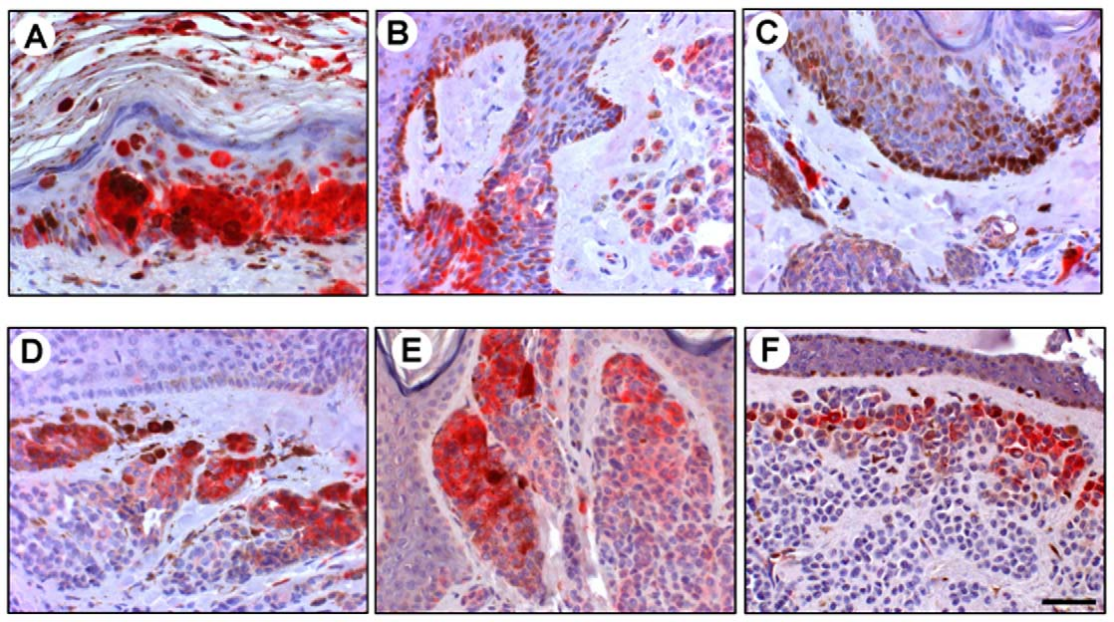
Expression of α-synuclein in human nevus tissues. Using mouse monoclonal anti-α-synuclein antibody 4D6, we immunostained tissue sections of 19 nevus samples (6 compound, 11 intradermal, and 2 junctional nevus samples) (see text for identification of samples). The sections were then incubated with an alkaline phosphatase-conjugated anti-mouse IgG. After washing, Liquid Permanent Red substrate was used to develop the reaction and detect α-synuclein-positive cells. The sections were then counterstained with hematoxylin and analyzed by microscopy. Six immunostainings are selected and shown in this figure. Panels **A**–**C**, compound nevus; Panels **D**–**F**, intradermal nevus. The localization of α-synuclein is shown by the red color of Liquid Permanent Red substrate. Nuclear counterstaining is shown by the blue color of hematoxylin. Melanin pigments are brown. Scale bar indicates 50 µm.

Fig. 7 shows α-synuclein immunostainings of 6 nevus samples, which are selected from the 19 immunostainings.

Panels A–C shows immunostaining of compound nevus. The section in panel A was derived from the right waist of a 23-year-old female. The section in panel B was derived from the left leg of a 25-year-old male. The section in panel C was derived from the upper arm of a 50-year-old male. In these samples, nevus cells are seen in the epidermis, and some have migrated into the dermis. In panel A, α-synuclein-positive nevus cells are mainly present as clusters at the junction between the dermis and epidermis. It should be noted that α-synuclein is immunostained in the stratum corneum, suggesting that α-synuclein is still stable in dead cells. In panel B, nevus cells strongly positive for α-synuclein occur at the junction between the dermis and epidermis, as seen in panel A. Some nevus cells in the dermis are also positive for α-synuclein. In panel C, some nevus cells are α-synuclein-positive in the dermis.

Panels D–F shows immunostaining of intradermal nevus. The section in panel D was derived from the face of a 42-year-old male. The section in panel E was derived from the rectum of a 38-year-old female. The section in panel F was derived from the anus of a 77-year-old male. These intradermal nevi show that nevus cells are entirely confined to nests in the dermis. Interestingly, nevus cells that are close to the epidermis are positive for α-synuclein, whereas nevus cells that are far from the epidermis are negative for α-synuclein.

Thus, α-synuclein is expressed in some non-malignant melanocytic nevus cells.

### α-Synuclein Is Undetectable in Non-melanocytic Cutaneous Carcinoma and Normal Skin

To examine the expression of α-synuclein in tissues of non-melanocytic cutaneous carcinoma, we performed α-synuclein immunostaining on tissue sections derived from 6 patients with squamous cell carcinoma and from 5 patients with basal cell carcinoma. For each patient, 3 sections were immunostained (total 18 sections of squamous cell carcinoma and 15 sections of basal cell carcinoma) (Table 4). Melanoma and normal skin sections were also immunostained on the same slide as positive and negative controls, respectively.

**Table 4:** Summary of α-synuclein immunoreactivity in non-melanocytic cutaneous carcinoma.

	α-Synuclein	
	Negative	Positive
**Squamous cell carcinoma: 6**	6 (100%)	0 (0%)
**Basal cell carcinoma: 5**	5 (100%)	0 (0%)
**Total: 11**	11 (100%)	0 (0%)

**Figure 8:**
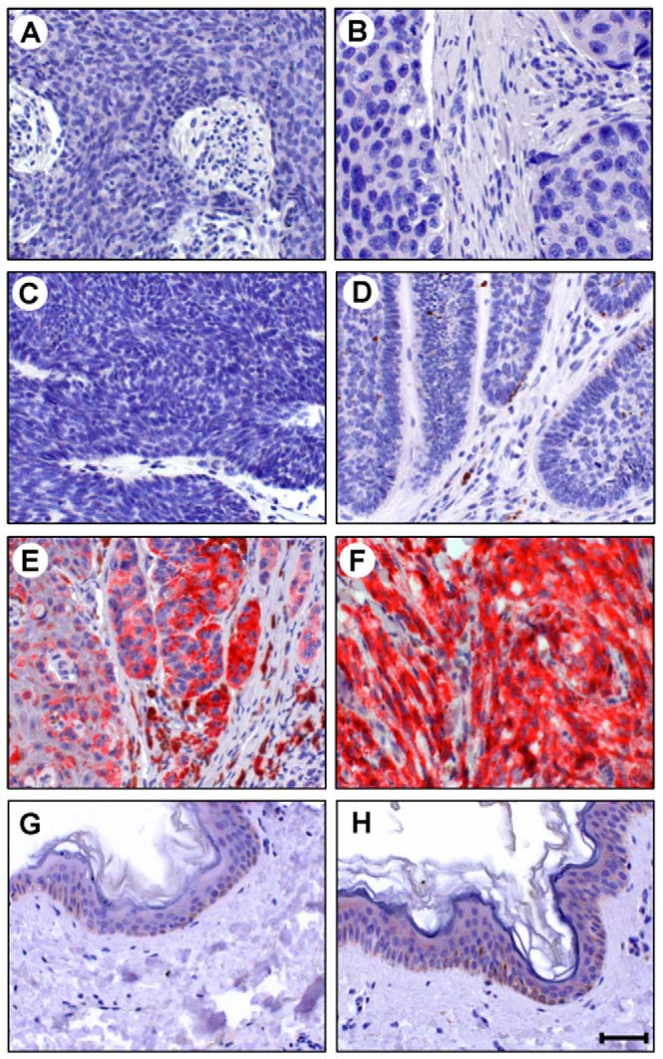
Expression of α-synuclein in tissues of patients with non-melanocytic cutaneous carcinoma. Using mouse monoclonal anti-α-synuclein antibody 4D6, we immunostained skin sections of squamous cell carcinoma (6 samples) and basal cell carcinoma (5 samples), as well as malignant melanoma (positive control) and normal skin (negative control). The sections were then incubated with an alkaline phosphatase-conjugated anti-mouse IgG. After washing, Liquid Permanent Red substrate was used to develop the reaction and detect α-synuclein-positive cells. The sections were then counterstained with hematoxylin and analyzed by microscopy. For each malignant disease or normal skin, two immunostainings were selected and shown in this figure. Panels **A** and **B**, squamous cell carcinoma; Panels **C** and **D**, basal cell carcinoma; Panels **E** and **F**, malignant melanoma; Panels **G** and **H**, normal skin. The localization of α-synuclein is shown by the red color of Liquid Permanent Red substrate. Nuclear counterstaining is shown by the blue color of hematoxylin. Melanin pigments are brown. Scale bar indicates 50 µm.

Fig. 8A and 8B show α-synuclein immunostainings of squamous cell carcinoma samples. The sections in panels A and B were derived from the chest skin of a 46-year-old male and a 73-year-old male, respectively. α-Synuclein was undetectable in these sections as well as the 16 other squamous cell carcinoma samples tested.

Fig. 8C and 8D show α-synuclein immunostainings of basal cell carcinoma samples. The section in panel C was derived from the submaxilla skin of a 63-year-old male. The section in panel D was derived from the skin of a 48-year-old male. α-Synuclein was undetectable in these sections as well as the 13 other basal cell carcinoma samples tested.

Fig. 8E and 8F show α-synuclein immunostainings of melanoma samples. The section in panel E was derived from the right thigh of a 66-year-old male. The section in panel F was derived from the left buttock of a 75-year-old female. α-Synuclein was strongly immunostained in both sections.

Fig. 8G and 8H show α-synuclein immunostainings of normal skin samples. The section in panel G was derived from the abdominal skin of a 40-year-old male. The section in panel H was derived from the breast skin of a 49-year-old female. Although brown melanin pigment was detected in the epidermis of both sections, α-synuclein was undetectable.

## Discussion

α-Synuclein is predominantly expressed in the brain. Point mutation or amplification of the α-synuclein gene causes PD, in which melanin-positive, dopaminergic neurons of the substantia nigra are degenerated [13]. Thus, α-synuclein is involved in the pathogenesis of PD, but its relationship with melanoma has been unknown.

Recent epidemiological studies have reported co-occurrence of PD and melanoma. Specifically, in a large-scale study including over 8,000 PD cases, a diagnosis of melanoma was associated with an approximately 50% increased risk of subsequently developing PD [10], whereas individuals with PD had a twofold increase in risk of subsequently developing melanoma [11]. Most recently, in another large-scale study including approximately 160,000 people who did not have PD, it was found that people with a family history of melanoma were nearly twice as likely to develop PD as people with no family history [12]. These epidemiological studies suggest that melanoma and PD could share common genetic components [12]. In this study, we demonstrated that both cell lines and tissues of malignant melanoma express α-synuclein. It is etiologically of interest that the PD-related protein, α-synuclein, is expressed in melanoma whose co-occurrence with PD has epidemiologically been reported.

MART-1 and HMB-45 are melanosome-related proteins [14], [15]. In this study, we demonstrated that melanoma cell lines positive for MART-1 and HMB-45 also express α-synuclein, suggesting that α-synuclein may be related to melanosomes or melanogenesis. Indeed, α-synuclein was previously shown to negatively regulate the activity of tyrosine hydroxylase [16], [17], the rate-limiting enzyme in the production of dopamine and melanin [18], [19]. In our immunohistochemical studies, melanoma cells highly expressing α-synuclein generate no or only very low levels of melanin pigments. In these cells, α-synuclein may inhibit the activity of tyrosine hydroxylase [16], [17] and affect melanin production as described previously [18], [19].

As discussed in the Introduction, S-100, HMB-45, and MART-1 are commonly used as histological biomarkers for malignant melanoma [4]. Because MART-1 staining has a higher diagnostic accuracy than S-100 and HMB-45 staining [4], [9], MART-1 has appeared to be the most useful histological biomarker for the diagnosis of melanoma. However, our histological studies using double immunostaining showed that α-synuclein expresses in MART-1-negative melanoma cells in many cases and that α-synuclein is positively immunostained in more melanoma cases than MART-1. Thus, α-synuclein might be a better histological biomarker for the diagnosis of malignant melanoma. Importantly, however, α-synuclein is expressed in both malignant and benign melanocytic lesions, such as melanoma and nevus, respectively. This means that α-synuclein cannot be used to distinguish between malignant and benign melanocytic skin lesions, but it is still very useful for the diagnosis of metastatic melanoma. Since α-synuclein is strongly expressed in unpigmented melanoma, as described above, this marker would allow us to easily diagnose unpigmented metastatic melanoma.

In conclusion, the PD-related protein, α-synuclein, is expressed in melanoma cells. Further testing of additional primary and metastatic tumor types will be required to evaluate the utility of α-synuclein immunostaining in the differential diagnosis of primary and metastatic melanoma in the clinical setting.
